# Multi-Tasking POM Systems

**DOI:** 10.3389/fchem.2018.00365

**Published:** 2018-08-21

**Authors:** Kevin P. Sullivan, Qiushi Yin, Daniel L. Collins-Wildman, Meilin Tao, Yurii V. Geletii, Djamaladdin G. Musaev, Tianquan Lian, Craig L. Hill

**Affiliations:** ^1^Department of Chemistry, Emory University, Atlanta, GA, United States; ^2^Emerson Center for Scientific Computation, Emory University, Atlanta, GA, United States

**Keywords:** Polyoxometalates (POMs), heterogeneous catalysis, multi-functional polymers, catalytic water oxidation, photoelectrochemical water splitting

## Abstract

Polyoxometalate (POM)-based materials of current interest are summarized, and specific types of POM-containing systems are described in which material facilitates multiple complex interactions or catalytic processes. We specifically highlight POM-containing multi-hydrogen-bonding polymers that form gels upon exposure to select organic liquids and simultaneously catalyze hydrolytic or oxidative decontamination, as well as water oxidation catalysts (WOCs) that can be interfaced with light-absorbing photoelectrode materials for photoelectrocatalytic water splitting.

## POM properties that make them effective in materials applications

Materials chemistry has evolved to the point where rational design of multifunctional systems with synergistic capabilities is possible. POMs are very effective as components of materials owing to their great synthetic tunability, allowing for many physical and chemical properties to be tailored to specific applications. Incorporating POMs into heterogeneous systems therefore allows for a bottom-up approach to the development of multifunctional materials (Miras et al., 2012; He et al., 2014; Zhang et al., 2014).

Polyoxometalates are useful in catalysis and other applications owing to their redox potentials, acidities, polarities, negative charge densities on surface oxygens and other parameters (Hill, 1998; Wang and Yang, 2015). Several reviews can be consulted for early studies of fundamental structure and reactivity (Pope, 1983, 2004; Hill, 2004; Yamase and Pope, 2006; Wang and Yang, 2015). The polyanion can be extensively modified by substitution of surface metal-oxo units (addenda or surface metal-oxo units) with many first-row transition-metal and other redox-active metal ions as well as organometallic groups. Of the large number of polyanion structural families, derivatives of the two most common families, Keggin and Wells-Dawson, still dominate fundamental studies of the impact of polyanion substitution and modification on the chemistry of POMs (Coronado and Gómez-García, 1995; Hill, 1998; Borrás-Almenar et al., 2001; Long et al., 2010). The fact that POMs are polyoxoanions with several counterions balancing the charge allows this huge and growing class of inorganic cluster compounds to be highly versatile; both the polyanion and the counterions can be altered, impacting the factors that make POMs useful in catalysis and other applications. This variability allows for small changes to be made in the POM systematically, facilitating the study and optimization of the resulting material.

The redox potentials of POMs are key to the reactions that feature prominently in many applications. POM potentials are controlled by the redox-active metals in the POM framework, by transition metal substituted into addenda (outside) structural sites, and by the charge density and geometry of the POM. Different POM geometries have intrinsically different charge densities on the framework metals and the oxygens bridging these metals, thus both the framework and substituted transition metals have altered potentials when present in different POM structural families. In addition to the framework metal and/or substituted transition metal, the nature and type of the counterion can also impact POM potentials (Hill, 2004). Ever more studies have demonstrated that POM counterions impact nearly every property of POMs that feature in their applications (Grigoriev et al., 2000, 2001).

The relative ease of incorporating POMs into heterogeneous matrices is another reason they are particularly well-suited for construction of functional materials. Several strategies exist for the immobilization of POMs including electrostatic attraction, solvophobic interactions, or covalent linkages (Proust et al., 2012; Hill and Kholdeeva, 2013; Xiao et al., 2016). Immobilization of POMs through electrostatic and solvophobic interactions is the most common and simple method, owing to the high negative charge of the POMs and ease of counter cation exchange (to produce insoluble salts)(Proust et al., 2012). Attaching POMs to materials covalently leads to the most stable products, however relatively few methods exist to achieve this covalent linkage while retaining all of the desired properties of the POM. As such, the development of covalently functionalized materials remains a very active area of research. Many studies have focused on the development of POM-based polymers to enable easier processing and facilitate the creation of POM-based devices and advanced materials. The tuning of flexible organic ligands and polyanions in POM-hybrids also allows for the engineering of POM-based compounds and materials with specific desired topologies (Taleghani et al., 2016). Most commonly, covalent incorporation of POMs and other inorganic compounds into polymer matrices is achieved through side-chain functionalization either before or after the polymerization process (Hu et al., 2012; Rieger et al., 2012; Macdonell et al., 2015; Wu et al., 2016). Both the self-assembly of POM-organic hybrids and their incorporation into polymers have been reviewed recently (Carraro and Gross, 2014; Wu et al., 2016).

## Survey of POM materials applications

**Figure 1:**
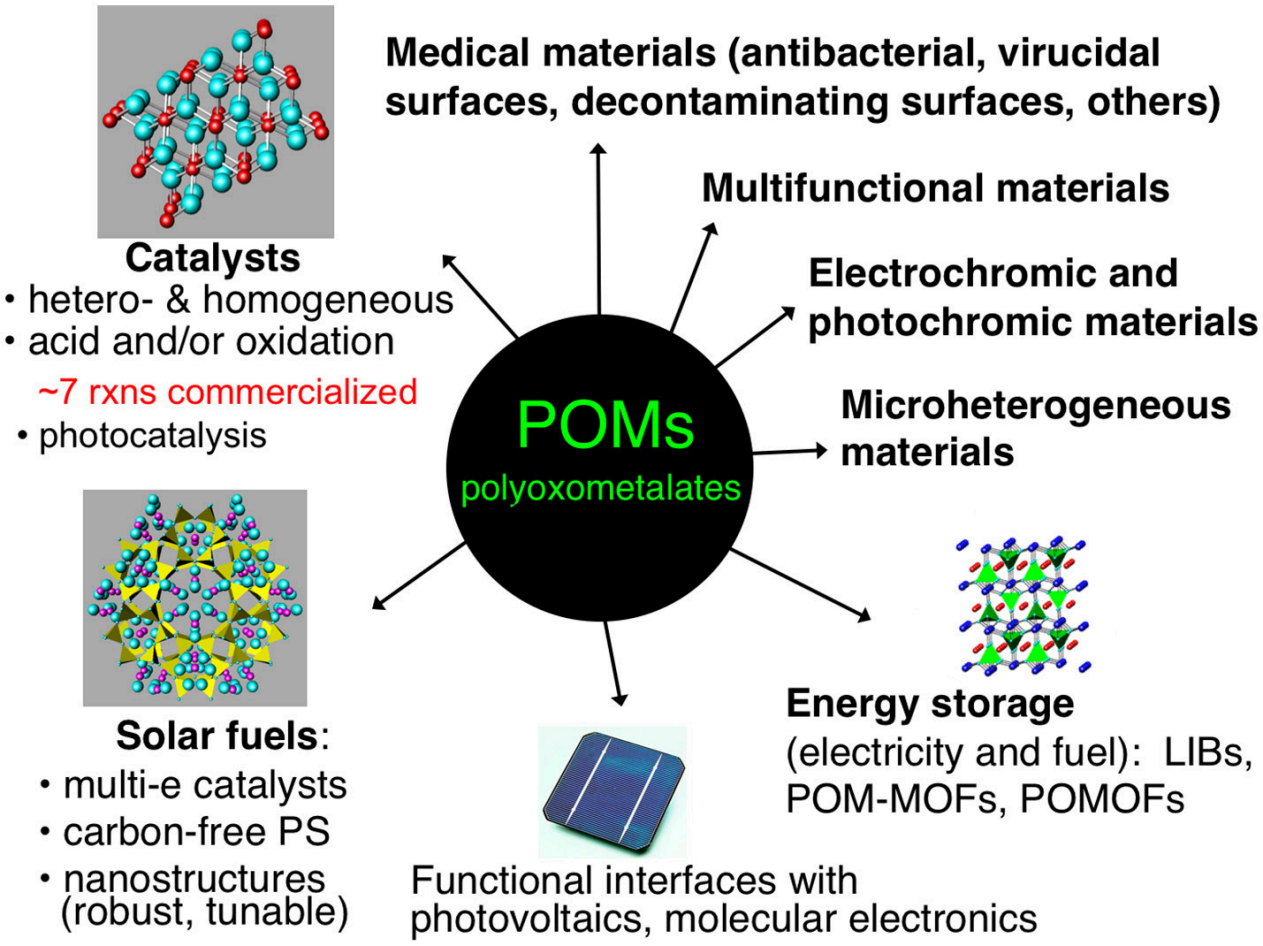
Some uses and ongoing research efforts involving POMs in materials.

Figure 1 illustrates several applications of POMs in materials. Some of these topics, such as catalysis of organic transformations, are more mature than others, but substantial research continues in all these areas. Several heterogeneous and homogeneous processes catalyzed by POMs have had commercial applications, such as in the hydration of alkenes or polymerization of tetrahydrofuran (Kozhevnikov, 1998). These industrial processes primarily involve the use of heteropolyacids as acid catalysts or more complex, mixed-metal POM derivatives as oxidation catalysts (Hill and Prosser-McCartha, 1995; Okuhara et al., 1996; Kozhevnikov, 1998, 2002; Mizuno and Misono, 1998; Moffat, 2001).

Several recent reviews have been published covering applications of POMs in heterogeneous catalysis (Long et al., 2007; Nlate Jahier and Jahier, 2012; Ren et al., 2015; Wang and Yang, 2015; Patel et al., 2016). One area of significant interest in POM-based materials has been the incorporation of POMs into metal-organic frameworks (MOFs) (Du et al., 2014). MOFs have attained a very high profile in the field of heterogeneous materials, as their high surface area and modifiable topologies allow for the design of a variety of very active functional materials (Corma et al., 2010; Furukawa et al., 2013). Combining the versatility of function of POMs with the high surface area of MOFs has led to several interesting and highly active materials. Song et al. reported the synthesis and characterization of a MOF (MOF-199) containing the Keggin-type POM [CuPW_11_O_39_]^5−^ within its pores. This POM-MOF exhibited substantially increased catalytic aerobic oxidation of sulfides and thiols to deodorized products compared to the POM or the MOF alone (Song et al., 2011). Ma et al. carried out a similar study in which a POM [PW_12_O_40_]^3−^ was used as a template during the synthesis of the MOF (NENU-11), aiding in its formation during a simple one-step hydrothermal synthesis. The resulting POM-MOF was then shown to be active towards the adsorption and hydrolysis of dimethyl methylphosphonate, an analog of organophosphonate chemical warfare agents, demonstrating synergistic roles of both the POM and the MOF in the composite material (Ma et al., 2011).

As mentioned previously, POM organic/inorganic hybrids are highly attractive for developing processable materials that incorporate the functionality of POMs. Research on POM organic/inorganic hybrids has developed into an extensive field with applications in many disciplines (Zonnevijlle and Pope, 1979; Gouzerh and Proust, 1998; Qi and Wu, 2009; Dolbecq et al., 2010; Proust et al., 2012; Song and Tsunashima, 2012). These include POM-modified organic/inorganic nanocomposites for energy applications (which will be discussed in greater depth later in this article)(Genovese and Lian, 2015; Ji et al., 2015), green synthesis(Omwoma et al., 2015; Zhou et al., 2015), photochemical and electrochemical properties (Walsh et al., 2016), environmental remediation (Sivakumar et al., 2012), among others. These POM materials often exhibit multiple synergistic functions. For example, Haimov et al. reported the synthesis of a cross-linked polyethyleneimine polymer containing [ZnWZn_2_(H_2_O)_2_(ZnW_9_O_34_)_2_]^12−^ POMs. The hydrophilic domains of the polymer contained both the POMs and the 2-alkanol substrates, and the lipophilic domain affects the solubility of the substrate in the hydrophilic domain. When the material was used to catalyze the hydrogen peroxide-based oxidation of the 2-alkanol substrates to the corresponding ketones, the material had the dual role of enhancing the reaction rate by bringing the POM and substrate in closer proximity and imparting a liposelectivity component to the reaction rate as a function of the hydrophobic nature of the substrate (Haimov and Neumann, 2006).

Another class of POM-based materials that warrant note are POMs intercalated into layered double hydroxides (LDHs). POM-LDH systems date back to Pinnavaia's early studies (Kwon et al., 1988), and have expanded to become an active field within POM materials chemistry (Omwoma et al., 2014; Li T. et al., 2017). An example by Zhao et al. demonstrates the synergistic effect of intercalating various sandwich-structure POMs into LDHs. They tested these materials on the mild and solvent-free oximation of aromatic aldehydes by the POM and observed substantial selectivity enhancements due to the ability of the LDH to suppress the formation of byproducts. Additionally, the heterogeneous support facilitated easy recovery and reuse of the material (Zhao S. et al., 2011).

Many types of POMs lend themselves to energy applications, such as energy storage and solar fuel generation. The ability of certain categories of POMs (most polytungstates, polymolybdates, and polyvanadates) to be reduced by many electrons, first dramatically noted in a study by Launay and co-workers (Launay, 1976) has led to intriguing studies of POM-based batteries and energy storage assemblies (Wang et al., 2012; Pratt et al., 2013; Genovese and Lian, 2015). One study published by Suárez-Guevara et al. uses electrodes made from activated carbon and the POM, H_3_PW_12_O_40_, in which the POM exhibits multiple functions: it increases the capacitance, operating voltage, and energy density of the battery. Additionally, the POM protects the activated carbon electrode from oxidation, allowing for the cell to have a high capacitance retention after a large number of charge-discharge cycles (Suárez-Guevara et al., 2014). An example of an energy application in which a POM has four planned and realized functions is a POM-Pt-MOF material that facilitates visible-light-driven catalytic H_2_ evolution and does this far more effectively than any of the 3 components alone. In this material, the POMs (1) catalyze reduction of platinum salts to Pt(0) nanoparticles, (2) stabilize the Pt nanoparticles and prevent them from aggregating, (3) induce a strong electrostatic association of the negatively charged Pt NPs with the protonated NH_2_-MIL-53 sites on the MOF particle surfaces, and (d) helps catalyze the H_2_ evolution reaction (Guo et al., 2016).

We now focus on two types of POM-based materials that illustrate the broad utility landscape of such materials. One involves the incorporation of a POM into the main chain of a polymer, resulting in a material which sequesters and decontaminates toxic or odorous compounds. The other involves immobilization of POM catalysts in photoelectrode assemblies in which the POM undergoes multiple photoinduced electron transfer processes and also carries the many steps involved in the oxidation of water molecules to O_2_.

## POM-containing network materials that physically entrap and catalyze degradation

**Figure 2:**
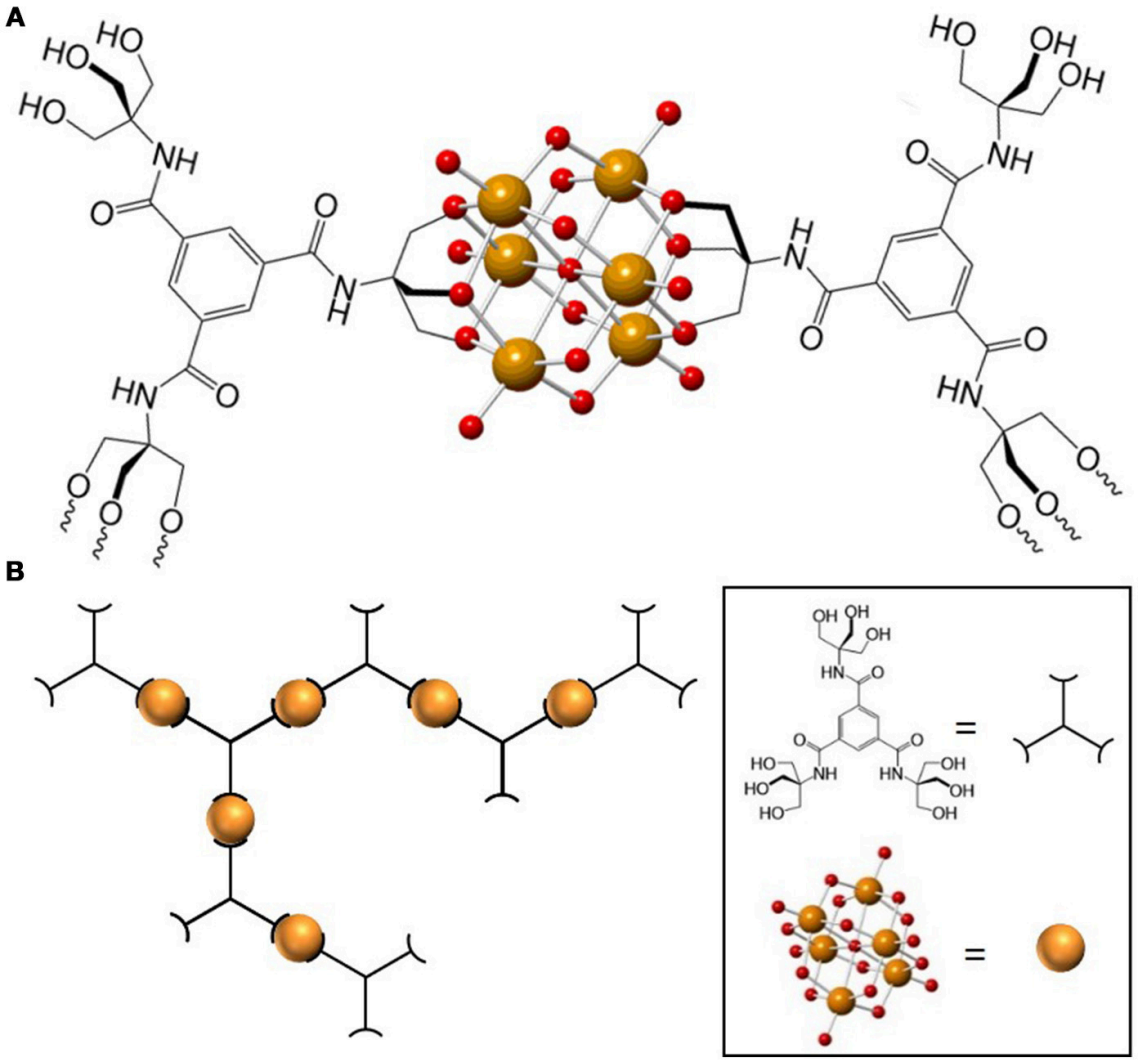
(**A**) Representation of a monomeric **TBA-polyV**_6_ unit. (**B**) Representation of the **TBA-polyV**_6_ polymer. Orange and red spheres represent V(V) and O^2−^, respectively. Reproduced from (Sullivan et al., 2017) with permission from the Royal Society of Chemistry.

Very recently we reported the design and synthesis of a POM-based polymer that demonstrates the advantages of combining the rich chemistry of POMs with properties obtained from incorporating the POM unit into a material. We synthesized and characterized a polymer composed of hexavanadate (V_6_O_19_) POM units and 1,3,5-benzenetricarboxamide-based linkers (trisBTA), with the formula [(*n*-C_4_H_9_)_4_N]_2n_[(V_6_O_13_)_n_[((OCH_2_)_3_CNHCO)_3_C_6_H_3_]_x_[((OCH_2_)_3_- CNHCO)_2_((HOCH_2_)_3_CNHCO)C_6_H_3_]_y_[(OCH_2_)_3_CNHCO ((HOCH_2_)_3_CNHCO) _2_C_6_H_3_] _z_] (**TBA-polyV**_6_, x = triply bound trisBTA, y = doubly-bound trisBTA, z = singly-bound trisBTA) (Figure 2)(Sullivan et al., 2017). This class of materials exhibits several functions: it forms gels within seconds after contact with polar aprotic organic liquids, catalyzes the oxidative or hydrolytic degradation of toxins and odorants under mild conditions, and exhibits color change during select oxidation reactions. This combination of properties was made possible through the incorporation of the POMs (capable of catalyzing oxidation and hydrolysis) and the organic linkers to form a polymeric material. The design of this polymer material from modular components allowed us to tailor the multiple functions to be useful for the entrapment and removal of toxic substances, including chemical warfare agent (CWA) analogs.

**Figure 3:**
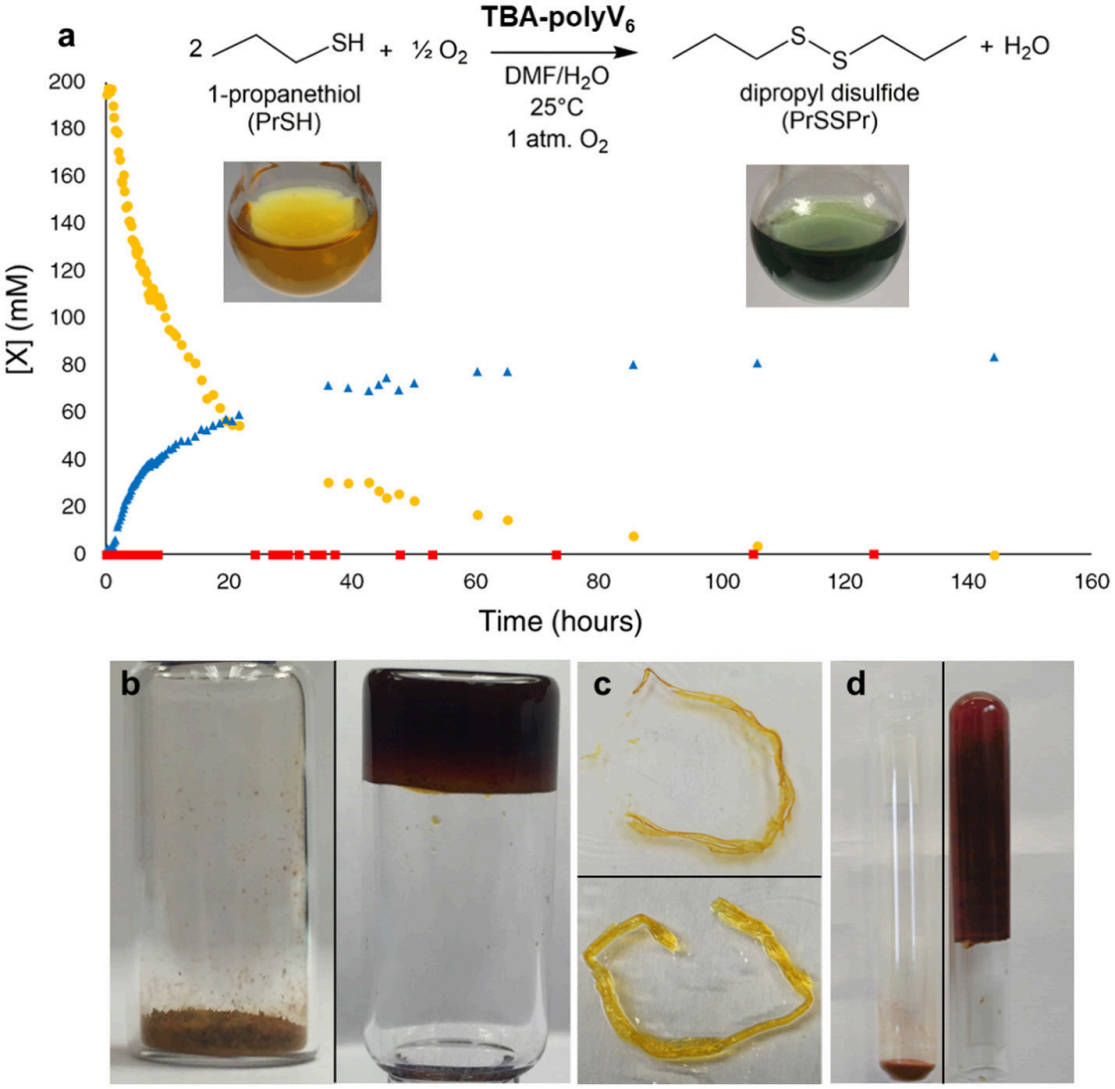
Aerobic oxidation catalyzed by **TBA-polyV**_6_. (**a**) Oxidation of 1-propanethiol (•) to dipropyl disulfide (▴) catalyzed by **TBA-polyV**_6_. The molar ratio was 127 1-propanethiol: 1 V_6_. A control reaction (■) was run under identical conditions but without **TBA-polyV**_6_. **Inset**: A yellow dispersion of **TBA-polyV**_6_ in DMF before (left) and after (right) addition of 1-propanethiol **(Figure 4.)** (**b**) Swelling of **TBA-polyV**_6_ (left) in the presence of dimethyl methyl phosphonate (DMMP) (right). **(c)** A strand of **TBA-polyV**_6_ before (above) and after (below) addition of DMSO. **(c)** Swelling of **TBA-polyV**_6_ (left) in the presence of DMF (right). **(d)** Swelling behavior measured in mL of liquid per gram of material after 24-hour exposure. Reproduced from (Sullivan et al., 2017) with permission from the Royal Society of Chemistry.

Polyoxometalate-organic hybrid species can oxidize a variety of organic substrates (Dolbecq et al., 2010), with hexavanadates demonstrating particular activity for oxidation of sulfides (Hill et al., 2006; Han and Hill, 2007). Thus, redox-active **TBA-polyV**_6_ facilitates color-change detection and oxidative decontamination. To demonstrate the applicability of **TBA-polyV**_6_ for air-based oxidative removal reactions, we conducted studies on the catalytic oxidation of 1-propanethiol (PrSH), as thiols are a major class of odorants in human environments. This representative thiol is fully oxidized to the corresponding non-odorous disulfide, and oxygen reoxidizes the reduced POM units (Figure 3a). The material is red in its powder state and reddish-orange when dispersed in solvent, indicative of the oxygen-to-metal charge transfer absorption manifold of a fully-oxidized [${\text{V}}_{6}^{\text{V}}$O_13_(OR)_6_)]^2−^ core (R = trisBTA linkers). Upon reduction of the POM, **TBA-polyV**_6_ becomes dark green as a broad peak between 600 and 900 nm increases, attributed to intervalence charge-transfer bands in the reduced POM (Chen et al., 1992). The persistent observation of a green reduced hexavanadate species during the reaction provides colorimetric detection capabilities for this polymeric material (Figure 3). In addition to aerobic oxidation of thiols, **TBA-polyV**_6_ catalyzes the oxidation of sulfides by hydrogen peroxide, including 2-chloroethyl ethyl sulfide, an analog of the CWA sulfur mustard. The sulfide is completely oxidized within 30 min after adding H_2_O_2_, whereas a reaction run without catalyst requires multiple hours to go to completion.

Recent studies have demonstrated both polyoxometalate-catalyzed hydrolysis of phosphoester bonds as well as hydrogen bond donor-catalyzed hydrolysis of organophosphate (OP) CWAs (Steens et al., 2010; Barba-Bon et al., 2013; Sambrook and Notman, 2013; Kinnan et al., 2014). Additionally, Zr-based MOFs and POMs have received a great deal of attention for their high activities toward hydrolysis of organophosphates, including OP nerve agents (Mondloch et al., 2015; Luong et al., 2016; Collins-Wildman et al., 2018). To demonstrate that **TBA-polyV**_6_ is highly modular in nature, we synthesized a new polymer through simple cation exchange with zirconyl chloride, affording **Zr-polyV**_6_, which exhibits high activity for catalytic hydrolysis of dimethyl *p*-nitrophenylphosphate (DMNP). We again show how the chemical and physical properties of the multifunctional POM-based polymer can be readily tuned to achieve targeted applications.

After demonstrating that the catalytic capabilities of the POM units remain intact in the heterogeneous material, we examined the gelation capabilities of the polymers. Studies of compounds containing 1,3,5-benzenetricarboxamides have shown them to be capable of forming gels through the presence of extensive hydrogen-bonding networks through the amide units, as well as π-stacking between adjacent aromatic groups (Sambrook and Notman, 2013). This property is preserved in **TBA-polyV**_6_, demonstrated by organogelation resulting from the addition of polar aprotic liquids to the material (Figures 3b–d). Significantly, we observed that **TBA-polyV**_6_ can form gels when exposed to OP agent analogs. Addition of the nerve agent analog dimethyl methyl phosphonate DMMP results in immobilization of a substantial amount of the liquid with rapid swelling kinetics. We demonstrate, therefore, that incorporating multiple functionalities to this POM-based polymer has allowed us to develop the first examples of materials that are potentially capable of both immobilizing and decontaminating CWAs.

## Heterogeneous polyoxometalate water oxidation catalysts (WOCs) and their use in photoelectrocatalytic water splitting

The applications of POMs or POM-based materials to solar fuels (artificial photosynthesis) is now a substantial category by itself. POMs have been examined in several functional roles for solar fuel generation. The first of these is broad-spectrum and intense visible light absorption with charge separation. Some POM derivatives have high extinction coefficients for charge transfer absorption (Zhao C. et al., 2011; Zhao et al., 2013), and while the charge-transfer excited-state lifetimes are highly variable, most are too short to result in high-quantum-yield chemical capture. However, POMs are quite active as catalysts for multi-electron reduction (reduction of H_2_O to H_2_ or CO_2_ to carbon-based fuel molecules)(Wang et al., 2016; Gumerova and Rompel, 2018). Several POMs that are efficient water reduction catalysts under visible-light-driven or dark conditions have been reported (Lv et al., 2014, 2015, 2016), and some POMs facilitate CO_2_ reduction under appropriate conditions (Ettedgui et al., 2010; Wang et al., 2016). One of the primary applications that has been the subject of extensive research within the field of POMs has been the catalysis of water oxidation. Since the original reports of a POM water oxidation catalyst (WOC), namely the tetra-ruthenium sandwich polytungstate, [{Ru_4_O_4_(OH)_2_(H_2_O)_4_}(γ-SiW_10_O_36_)_2_]^10−^ (Ru_4_Si_2_)(Geletii et al., 2008; Sartorel et al., 2008), and the first publication on a POM WOC of all earth-abundant elements (Yin et al., 2010), there have been scores of papers on POM WOCs (Lauinger et al., 2017b; Li J. et al., 2017).

The issue of the true active catalyst species is always one of great interest in any catalytic system (Vickers et al., 2013; Wu et al., 2015). Polyoxometalate WOCs have been of particular concern in this regard (Stracke and Finke, 2011, 2014; Vickers et al., 2013; Folkman and Finke, 2017). This is due to the fact that POMs are essentially molecular metal oxides and could potentially have extensive speciation equilibria in solution (Sumliner et al., 2014; Sara et al., 2015; Lauinger et al., 2017b; Nyman, 2017). Some of the species that could be present include the metal aqua complexes as well as the corresponding metal oxide nanoparticles, all of which could also be active as WOCs (Stracke and Finke, 2014; Sumliner et al., 2014; Folkman and Finke, 2017; Lauinger et al., 2017b; Suen et al., 2017). It is therefore essential that any POM species is demonstrated to be stable and active under experimental conditions. POMs have been immobilized on photoanodes to make photocatalytic water oxidizing electrodes, allowing careful tailoring of POMs during their synthesis to be combined with the stabilizing effects of a heterogeneous material. Heterogeneous systems involving POMs are generally more stable than their molecular counterparts due to the limiting of solution phase equilibria involving the POM, preventing speciation that could lead to a shift in the composition of the catalytic POM cluster (Lauinger et al., 2017b). Heterogeneous WOCs are also advantageous because both half reactions involved in water splitting are spatially separated, preventing product recombination or the collection of an explosive mixture of hydrogen and oxygen. In addition, heterogeneous architectures enable efficient charge transfer and separation between photosensitizer and catalyst. In a homogeneous setup, the two components must diffuse to one another leaving time for charge recombination or breakdown of unstable intermediates, lowering the overall system efficiency.

**Figure 4:**
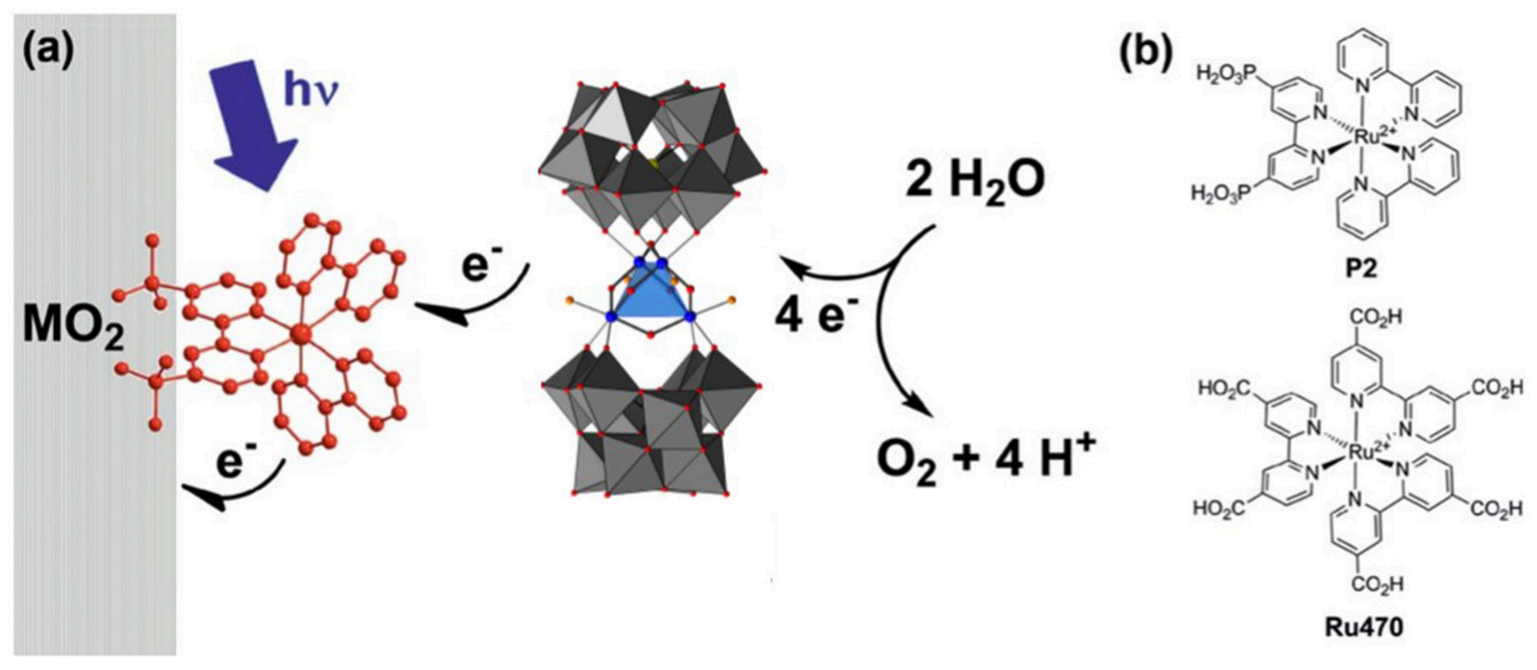
**(a)** Principle of operation of a triadic water-oxidizing photoanode incorporating both Ru_4_Si_2_ and a dye. **(b)** Structures of the dyes which have been used in triads with Ru_4_Si_2_ (Xiang et al., 2013). Adapted with permission from J. Phys Chem. C., 2013, 117 (2), pp 918–926. Copyright 2013, American Chemical Society.

In general, studies of POMs immobilized on photoelectrodes are of three types: electrostatic binding to a surface-bound photosensitizer dye, electrostatic immobilization on semiconductor photoanodes, and partial encapsulation of POM WOCs on photoanodes via metal oxide nanofilm deposition. Studies done by Xiang et. al. used ultrafast transient absorption spectroscopy to probe electron transfer between the Ru_4_Si_2_ WOC, photosensitizer dye [Ru(bpy)_2_(dpbpy)]^2+^, and various metal oxide electrode surfaces (Figure 4) (Xiang et al., 2013). A similar study was also reported by Bonchio, Scandola and co-workers in which illumination of the POM-dye-metal oxide triad resulted in a rapid (10 ns) electron injection which occurs from the catalyst to sensitizer, generating a long lived (0.5 μs) charge-separated state (Orlandi et al., 2010). This proved to be an effective strategy for water oxidation as Fielden et. al. later demonstrated with oxygen measurements on a similar triad (Fielden et al., 2015), however, a limitation in both cases was the stability of the dye on the surface.

**Figure 5:**
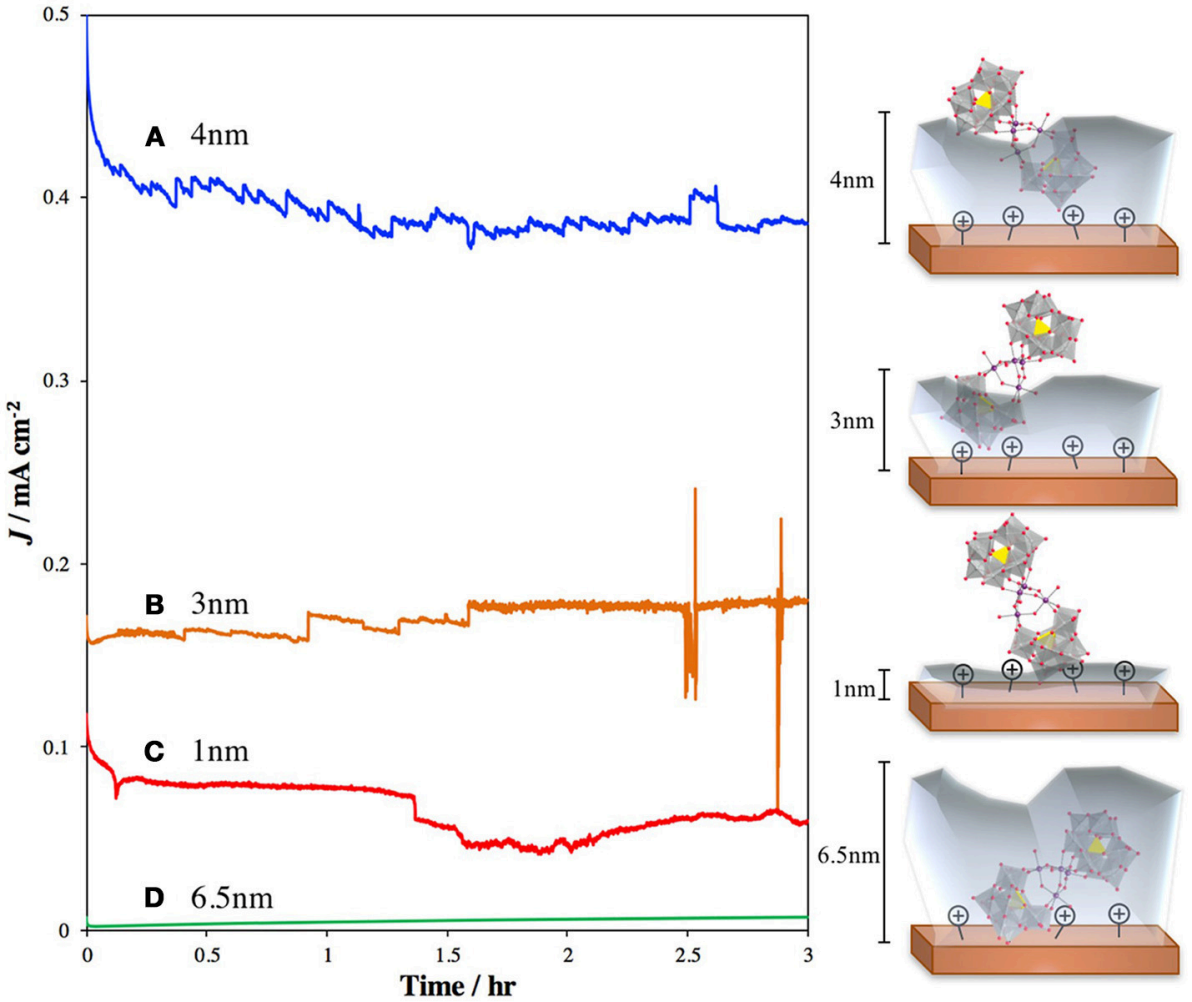
Current densities and schematic illustrations of photoanodes composed of hematite–APS–Ru_4_Si_2_-Al_2_O_3_ atomic layer deposition thickness of **(A)** 4 nm (blue line); **(B)** 3 nm Al_2_O_3_ (orange line); **(C)** 1 nm Al_2_O_3_ (red line); and **(D)** 6.5 nm Al_2_O_3_ (green line) (Lauinger et al., 2017a). The translucent gray layers represent the depth of the Al_2_O_3_ coating.Reprinted with permission from *ACS Appl. Mater. Interfaces*, **2017** 9 (40), 35048–35056. Copyright 2017, American Chemical Society.

Several studies have focused on direct binding of the POM to photoactive metal oxides, such as TiO_2_ and hematite. Recently, our group published a report in which we treated TiO_2_ with 3-aminopropyltrimethoxysilane (APS) to generate a cationic surface that strongly bound the highly negatively charged POM WOC, Ru_4_Si_2_ (Lauinger et al., 2015). This was effective for surface immobilization of the POM and light driven water oxidation. To improve this system by moving the observed catalysis from the UV into the visible range, we examined surface treatment of hematite with Ru_4_Si_2_ as the POM WOC to achieve visible-light-driven water oxidation (Lauinger et al., 2017a). In this system, atomic layer deposition (ALD) of Al_2_O_3_ was used to provide stabilization through partial encapsulation of the POM. The thickness of the ALD layer was optimized to prevent desorption of the catalyst without greatly reducing the efficiency of electron injection from Ru_4_Si_2_ into the hematite electrode (Figure 5).

Perhaps most impressive of recent efforts to turn known homogeneous POM WOCs into functional heterogeneous electrocatalysts involves the immobilization of a septa-cobalt POM into a graphite matrix using a carbon paste electrode (Blasco-Ahicart et al., 2017). By formulating the POM cluster in combination with Ba^2+^ and a stable graphite matrix, Blasco-Ahicart et al. observed a dramatic synergistic effect of both increased stability and catalytic performance. This was the first reported example of a stable discrete complex with earth-abundant elements for water oxidation in strongly acidic media. At certain current densities, this heterogeneous composition even manages to have a lower overpotential than the long-running state-of-the-art iridium oxide and ruthenium oxide WOC films. Having a concrete understanding of how this type of synergistic effect between the hydrophobic carbon paste framework and the POM occurs could yield substantial improvements in the WOC field (Yin and Hill, 2017).

## Conclusions

The field of POM-based materials has been expanding rapidly and is likely to remain fruitful as ever more applications are explored. The ease of tunability and high activity of POMs combined with the processability, stability, or other physical advantages of heterogeneous systems allows for extraordinary versatility in application of these materials. These efforts have led many scientists traditionally working on homogeneous systems towards materials chemistry, and vice versa. Thus, there is an imperative to combine the vast knowledge base of each of these fields and foster collaboration to aid in the production of high quality research towards the development and characterization of new hybrid POM systems.

## Author contributions

KS and CH oversaw all writing. QY, DC-W, MT, YG all contributed to the writing of the WOC section. DM and TL provided input.

### Conflict of interest statement

The authors declare that the research was conducted in the absence of any commercial or financial relationships that could be construed as a potential conflict of interest.
